# Effectiveness of time-limited eye movement desensitization reprocessing therapy for parents of children with a rare life-limiting illness: a randomized clinical trial

**DOI:** 10.1186/s13023-022-02500-9

**Published:** 2022-09-02

**Authors:** T. Conijn, C. De Roos, H. J. I. Vreugdenhil, E. M. Van Dijk-Lokkart, F. A. Wijburg, L. Haverman

**Affiliations:** 1 Emma Children’s Hospital, Amsterdam UMC, Child and Adolescent Psychiatry and Psychosocial Care, Amsterdam Reproduction and Development, Amsterdam Public Health, University of Amsterdam, Amsterdam, The Netherlands; 2 Emma Children’s Hospital and Amsterdam Lysosome Center “Sphinx”, Amsterdam UMC, Pediatric Metabolic Diseases, University of Amsterdam, Meibergdreef 9, Amsterdam, Netherlands; 3 Academic Center for Child and Adolescent Psychiatry, Amsterdam UMC, Levvel, University of Amsterdam, Amsterdam, The Netherlands

**Keywords:** PTSD, Trauma, Parents, EMDR, Life-limiting illness

## Abstract

**Background:**

Parents of children with a rare progressive life-limiting illness are at risk for parental posttraumatic stress disorder (PTSD). Studies on the treatment of parental PTSD with eye movement and desensitization reprocessing (EMDR) therapy in pediatric practice are lacking. Therefore this study aims to evaluate the feasibility and effectiveness of time-limited EMDR therapy in reducing PTSD symptoms, comorbid psychological symptoms, distress, and parental stress.

**Methods:**

Mono-center randomized clinical trial conducted between February 2020 and April 2021. Fourteen parents (*N* = 7 mothers, *N* = 7 fathers) of mucopolysaccharidosis type III patients reporting PTSD symptoms on a (sub)clinical level were assigned to EMDR or a wait-list control condition followed by EMDR. Four sessions of EMDR (each 90 min) divided over two half-days were offered. Measurements were conducted at baseline, post-treatment/post-waitlist, and 3-months post-treatment. The primary outcome was PTSD symptom severity (PTSD Check List for DSM-5). Secondary outcomes included comorbid psychological symptoms (Brief Symptom Inventory), distress (Distress Thermometer for Parents) and parenting stress (Parenting Stress Questionnaire). Between-group comparisons pre-to-post treatment (*N* = 7 EMDR vs. *N* = 7 wait-list) and within-group comparisons (EMDR, *N* = 14) from pre-to-post treatment and from pre-treatment to 3-months follow-up were carried out per intent-to-treat linear mixed model analyses.

**Results:**

Compared to wait-list, EMDR resulted in a significant reduction on total PTSD symptom severity (*d* = 1.78) and on comorbid psychological symptoms, distress and parenting stress (*d* = .63–1.83). Within-group comparisons showed a significant effect on all outcomes at post-treatment (*d* = 1.04–2.21) and at 3-months follow-up (*d* = .96–2.30) compared to baseline. EMDR was well-tolerated, associated with a low drop-out rate, a high therapy adherence and no adverse events.

**Conclusion:**

Time-limited EMDR reduces PTSD symptoms, psychological comorbidity, distress and parenting stress in parents of children with a rare progressive life-limiting illness. This treatment was feasible for these overburdened parents. Recurrent monitoring of PTSD symptoms, and, if needed, offering this time-limited type of trauma treatment should be introduced in everyday pediatric practice.

*Trial registration* Netherlands Trial Register, NL8496. Registered 01-04-2020, https://trialsearch.who.int/Trial2.aspx?TrialID=NL8496.

**Supplementary Information:**

The online version contains supplementary material available at 10.1186/s13023-022-02500-9.

## Introduction

Parenting a child with a progressive, life-limiting illness makes parents vulnerable for psychosocial problems [1, 2]. Their caregiver role, often consisting of intensive care on daily basis, results in a reduction of time for e.g., employment, social life and leisure activities [3]. In addition, parents are exposed to an accumulation of potential traumatic medical events during their child’s disease course and they have to deal with persistent fears about the future and feelings of loss [4, 5]. Exposure to ongoing stressful events may lead to a wide variety of psychological problems, including posttraumatic stress disorder (PTSD) [6]. According to the DSM-5, PTSD includes clusters of symptoms of intrusions (cluster B; e.g., nightmares), avoidance (cluster C; e.g., avoiding certain people or places), negative alterations in mood and cognitions (cluster D; e.g., feelings of guilt), and hyperarousal (cluster E; e.g., irritability) [7]. When parents experience these symptoms related to the traumatic experiences of their child and fulfill the criteria for PTSD it is referred to as parental PTSD. The model of Pediatric Medical Traumatic Stress (PMTS) describes these stress reactions as ‘’a set of psychological and physiological responses of children and their families to pain, injury, serious illness, medical procedures and invasive or frightening treatment experiences’’ that may evoke at several points in the course of a disease. The symptoms of parents or families in reaction to medical trauma does not always meet the requirements for the diagnosis PTSD. Therefore, PMTS is not conceptualized as a traumatic stress disorder, but as posttraumatic stress symptoms (PTSS) necessitating treatment without meeting the criteria for a PTSD diagnosis [8].

An illustration of a progressive, life-limiting illness which evoke such stress responses is mucopolysaccharidosis type III (MPS III or Sanfilippo syndrome). MPS III is a rare inherited metabolic disease characterized by progressive cognitive deterioration from approximately 2 years of age, often with severe behavioral problems, ultimately leading to a premature demise [9, 10]. No disease modifying treatment is available to date [11]. The majority of parents of children with MPS III demonstrate high levels of PTSD symptoms related to their child’s illness [12].

Effective treatment of parental PTSD is essential as chronic distress in parents has a negative impact on the cognitive, social and physical development of the child [13, 14]. Eye movement desensitization and reprocessing (EMDR) therapy is an effective treatment for PTSD [15, 16], and is recommended as first line treatment in clinical guidelines [17, 18]. The core principle of EMDR is that patients need to focus on emotionally disturbing memories (image, thoughts, emotions and sensations) while simultaneously focusing on a distracting visual or tactile stimulus. Due to the competition of working memory tasks, the traumatic memory representation become less vivid and emotionally disturbing and dysfunctional cognitions associated with the traumatic memory can be changed into more adaptive cognitions [19]. More than 30 RCT’s demonstrated the efficacy of EMDR in adults with PTSD. Most of these studies included patients who had experienced accidents, a natural disaster, war, sexual or physical maltreatment [16, 20]. Less studies have been conducted to assess the effectiveness of EMDR for patients with symptoms related to medical trauma. Regarding this topic, two previous studies showed that EMDR is a successful PTSD treatment for e.g. Multiple Sclerosis- and cancer patients [21, 22]. Studies on the effectiveness of EMDR for parental PTSD related to the disease of their child are lacking.

EMDR is generally offered in weekly sessions (60–90 min per sessions) over several weeks to months. Parents of children with a life-limiting illness are burdened by the extensive care for their child and they often live relatively far away from expertise centers, which makes frequent hospital visits difficult. In a recent case study, we demonstrate that a time-limited course of EMDR (four sessions of 90 min EMDR scheduled over two half-days) in two parents of MPS III patients resulted in a significant decrease of PTSD symptoms and psychological comorbidity [23]. The current randomized clinical trial aims to validate the feasibility and effectiveness of time-limited EMDR in parents of a child with a rare life-limiting illness in terms of reducing PTSD symptom severity, comorbid psychological symptoms, distress, and parenting stress.

## Methods

### Design

The study was a mono-center randomized clinical trial in which participants were randomly assigned to EMDR or to a wait-list control condition of six weeks, followed by EMDR (delayed treatment). The study was conducted in accordance with the Declaration of Helsinki (2013), registered in the Dutch Trial Register (NL8496) and approved by the Medical Ethics Committee of the Amsterdam University Medical Centers, location AMC. All parents gave written informed consent.

### Participants, procedure and randomization

All parents of a living child with MPS III known in the Amsterdam University Medical Centers, the only expertise center for MPS III in the Netherlands, received an information letter between February 2020 and April 2021. Inclusion criteria were: (1) parents meeting the criteria indicative for a PTSD diagnosis on the PTSD Check List for DSM-5 (PCL-5) or parents experiencing PTSS with at least a moderate or high score (2–4) on one symptom in each PTSD cluster or met 3 of the 4 PTSD criteria (one B symptom, one C symptom, two D symptoms and two E symptoms) measured by the PCL-5 or a subclinical score (> 24) on the PCL-5, and (2) sufficient knowledge of the Dutch language to complete the assessments. Exclusion criteria were: (1) major interfering acute medical or psychiatric condition, such as psychosis or high risk for suicide and (2) receiving psychological trauma treatment by another therapist during study participation. Participating parents completed online questionnaires to screen for eligibility (T0, baseline measurement). Parents who were part of a couple could both participate and followed an individual, independent trajectory in the study.

Eligible parents were randomly assigned on a 1:1 basis to one of the two study arms. Block randomization, with randomly selected block sizes of 2, 4 and 6, were used to reduce bias and achieve minimal differences in group sizes. An independent researcher performed randomization by computer. Parents and therapists were aware of the allocated arm, outcome assessors were kept blind.

Online validated questionnaires were completed at baseline/pre-treatment (T0), 2-weeks post-treatment (T1), and 3-months post-treatment (T2). The wait-list control group completed an additional questionnaire post wait-list (T.01) before receiving EMDR. After the last follow-up a telephonic interview was done to assess if parents considered the EMDR sufficiently successful or if referral for more psychological support was needed.

### Intervention

#### EMDR

EMDR therapy was offered to parents in the Amsterdam University Medical Center in Amsterdam. The intervention consisted of an intake session (first visit; 90 min) followed by four sessions EMDR (90 min per session) over two treatment days (two sessions per a half-day, 30 min between sessions). Time between the treatment days was one to two weeks. EMDR was offered by following the standard eight-phase protocol, consisting history taking and treatment planning, client preparation, assessment, desensitization, installation, body scan, closure and reevaluation of the treatment effect [24, 25]. Based on the EMDR targets that were identified in the case report of Conijn et al. [23], the therapists discussed the following themes during the intake session: the pre-diagnostic phase, the moment of diagnosis, progression of the disease, treatment and/or participation in experimental trials, negative experiences with medical day-cares, special education or residential nursing homes, social/family life, and their fears about the future. The intake session resulted in a standardized case conceptualization in which a hierarchy of stressful memories or flash forwards (a mental representation of a feared catastrophe) related to the disease of the child was listed and rated with the Subjective Units of Disturbance (SUD) score (0–10). The identified memories were placed in a hierarchy from high to low SUD and treated accordingly. During the EMDR sessions, parents had to focus on the emotionally disturbing memory, while simultaneously concentrating on a distracting stimulus for about 30 s. The parent reported briefly what comes up and was guided by the therapist to refocus attention to the memory along with the stimulus. Processing continued until the parent reported no remaining disturbance (SUD related to the memory) anymore. Then, a positive cognition was installed and residual disturbing body sensations were identified and processed. Finally, a positive closure took place and in the subsequent session the treatment effect was reevaluated. When the SUD’s of all memories that were part of the case conceptualization were 0, EMDR therapy was considered complete.

Treatment was provided by three EMDR-therapists, all licensed psychologists who had completed accredited courses in EMDR under monthly supervision. All EMDR sessions were audio or video-taped. A total of 10% (*N* = 5) of the treatment sessions was randomly selected, stratified on therapist and session, to be rated on treatment adherence by using an EMDR integrity checklist. Treatment adherence was 94%.

#### Wait-list

Parents in the wait-list condition did not receive any form of psychological support. They were aware that they could receive EMDR therapy after the waiting-list period.

### Measures

Socio-demographic characteristics and information on use of medication and psychological treatment were collected by self-report questionnaires.

#### Primary outcome

PTSD symptom severity was measured with the PTSD Check List for DSM-5 (PCL-5) [26], a self-report questionnaire that measures the 20 DSM-5 PTSD symptoms on the following subscales: intrusions (B), avoidance (C), negative alterations in cognitions and mood (D) and hyperarousal (E) over the last week. Items are rated on a five-point Likert-scale, ranging from 0 ‘not at all’ to 4 ‘extremely’ (total score range 0–80). A cut-off score of 33 is indicative for PTSD [27]. A cut-off score of 24 was considered as subclinical PTSD. The PCL-5 demonstrated adequate psychometric properties [27]. Cronbach’s alpha in this study is .89.

#### Secondary outcomes

Comorbid psychological symptoms were measured with the Brief Symptom Inventory (BSI) [28], a self-report questionnaire (53 items) consisting of a total score and nine subscales: somatization, obsessive–compulsive, interpersonal sensitivity, depression, anxiety, hostility, phobic anxiety, paranoid ideation and psychoticism. Items were measured on a five-point Likert scale from 0 = ‘none’ to 4 = ‘a lot’. The BSI demonstrated adequate psychometric properties [29]. Cronbach’s alpha is .94.

The Distress Thermometer for Parents (DT-P) was used to assess distress and everyday problems when parenting a chronically ill child [30]. The DT-P consists of a thermometer score measuring overall distress (0 = ‘no distress’ to 10 = ‘extreme distress’), accompanied by a problem list (divided over six domains: practical, family/social, emotional, physical, cognitive, and parenting (child’s age > 2 years version). Problem domain scores were the sum of the dichotomous items (0 = ‘no’ and 1 = ‘yes’) in each problem domain and a total problem score can be calculated. The DT-P demonstrated adequate psychometric properties [30]. Cronbach’s alpha is .85.

Parenting stress is measured with the Parenting Stress Questionnaire (OBVL), a self-report questionnaire consisting of 34 items that measures stress that parents may experience in five parenting domains: parent–child relationship, parental incompetence, depressed mood, health complaints and role restriction [31]. Items were measured on a Likert-scale ranging from 1 = ‘does not apply’ to 4 = ‘applies completely’. The Parenting Stress Questionnaire demonstrated adequate reliability and validity [31]. Cronbach’s alpha is .90.

### Statistical analyses

Statistical Package for Social Sciences (SPSS) version 26.0 for Windows was used for all statistical analyses (SPSS, Inc., Chicago, IL, USA). *p *values < .05 were considered statistically significant. Baseline differences between groups were analyzed using independent sample *t*-tests for continuous data and Fishers exact tests for categorical data.

Between-group comparisons (*N* = 7 EMDR vs. *N* = 7 waitlist control) at T1/T.01 on the primary outcome and the secondary outcomes were performed using intention-to-treat Linear Mixed Models (LMM) to account for dependency of data within participants and missing data. We did not account for dependency within families given the intraclass correlation coefficient of .15 and the small numbers within families [32]. The model was fitted with a random intercept and fixed slopes for intervention, time, and the interaction term intervention x time.

Within-group comparisons of the combined group to assess the effect of EMDR (*N* = 14) on the primary outcome and the secondary outcomes were performed using intention-to-treat LMM with time as fixed effect. Outcomes of the EMDR group at T0 and of the wait-list control group at T.01 were merged and used to describe the baseline measurement of the total group. The outcome of this baseline measurement was compared with the outcomes of the total group at T1 and T2. Between- and within effect sizes (Cohen’s *d)* were calculated, were < .49 was considered small, between .50 and .79 medium, > .80 large [33] (see Additional file 1: Supplementary material A for calculation of the effect sizes).

Finally, drop-out rates and adverse events were described to explore feasibility.

## Results

### Inclusion, attrition and sociodemographic and clinical characteristics

**Fig. 1:**
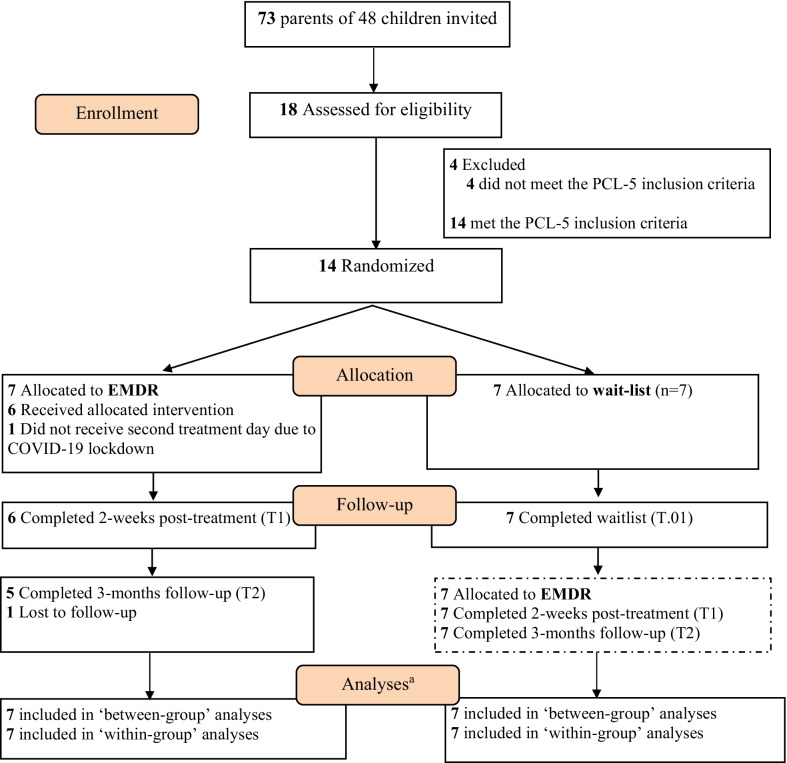
Consort diagram showing the flow of participants through the trial in each group. ^a^Analyses included all available data from each subject up to withdrawal or trial completion

Eighteen out of 73 invited parents completed the first set of questionnaires (response rate 19.2%). Fourteen parents (77.8%) met the inclusion criteria (*N* = 7 EMDR, *N* = 7 wait-list, see Fig. 1). Four parents were excluded as they did not report PTSD symptoms on a (sub)clinical level. No significant differences at baseline were found between the experimental and control group (Table 1). In total, three parent couples participated in the study.

**Table 1:** Baseline sociodemographic and clinical characteristics of intent-to-treat sample

	EMDR		WL		*p*
	*N*	*M* (*SD*)	*N*	*M* (*SD*)	
Demographic characteristics					
Age parent (years)	7	44.1	7	47.9	.49
Age child with MPS III (years)	7	12.4 (10.8)	7	15.9 (7.8)	.50

	N	%	N	%	
Gender (female)	3	42.9	4	57.1	.59
Married/living together	5	71.4	6	85.7	.52
Number of children living at home					.84
1	2	28.6	3	42.9	
2–3	4	57.1	3	42.3	
> 3	1	14.3	1	14.3	
Number of children with MPS III					1.0
1	6	85.7	6	85.7	
2	1	14.3	1	14.3	
Educational level^a^					1.0
Low	0	0.0	0	0.0	
Intermediate	3	42.9	3	42.9	
High	4	57.1	4	57.1	
Country of birth (the Netherlands)	6	85.7	6	85.7	1.0
Paid employment	5	71.4	5	71.4	1.0
Clinical characteristics					
Earlier psychological treatment	4	28.6	5	35.7	.50
Medication (antidepressant)	1	14.3	1	14.3	1.0

*WL* wait-list, *EMDR* eye movement and desensitization and reprocessing. ^a^Educational level: low (primary education, lower vocational education, lower and middle general secondary education), intermediate (middle vocational education, higher secondary education, pre-university education), and high (higher vocational education, university)

### Between-group comparisons (EMDR vs. wait-list)

#### Primary outcome

Compared to wait-list, EMDR resulted in a significant reduction on total PTSD symptom severity (PCL-5) (*d* = 1.78) at T1/T.01 (Table 2). Moreover, participants in the EMDR group reported a significant reduction on all subscale scores (*d* = 1.04–1.61) at T1/T.01.

**Table 2:** Means and standard deviations pre-treatment (T0), post-treatment/post-waitlist (T1/T.01), and between-group comparisons (EMDR vs. wait-list)

	EMDR		WL		Intervention × time		
	T0 (*N* = 7)	T1 (*N* = 6)	T0 (*N* = 7)	T0.1 (*N* = 7)			
	M (*SD*)	M (*SD*)	M (*SD*)	M (*SD*)	*B* [CI 95%]	*p*	*d*
PCL-5							
Total score	41.57 (13.40)	20.67 (15.00)	38.28 (13.33)	38.86 (11.63)	− 22.98 [− 32.77; − 13.19]	**< .001**	1.75
Cluster B	9.29 (2.69)	3.50 (2.59)	11.15 (3.57)	10.14 (3.57)	− 5.08 [− 7.48; − 2.67]	**.001**	1.61
Cluster C	4.86 (2.73)	2.00 (2.28)	4.71 (1.79)	4.00 (1.63)	− 2.40 [− 4.69; .11]	**.04**	1.04
Cluster D	13.57 (6.16)	7.50 (6.41)	11.14 (6.04)	13.28 (4.92)	− 8.83 [− 12.86; 4.80]	**< .001**	1.45
Cluster E	13.86 (3.67)	7.67 (4.23)	11.28 (6.52)	11.43 (4.43)	− 6.55 [− 10.64; − 2.47]	**.004**	1.24
BSI							
Total score	.97 (.47)	.50 (.36)	1.15 (.57)	1.03 (.55)	− .33 [− .63; − .28]	**.035**	.63
DT-P							
Thermometer score	7.14 (2.04)	3.67 (2.88)	6.00 (3.00)	7.29 (1.11)	− 4.70 [− 7.39; − 2.00]	**.002**	1.83
Total problem score	21.86 (5.70)	12.17 (8.26)	19.00 (7.70)	20.00 (5.03)	− 11.22 [− 15.63; − 6.81]	**< .001**	1.66
OBVL							
Parenting stress total	89.86 (9.67)	73.00 (12.82)	84.14 (17.01)	84.14 (13.21)	− 18.24 [− 25.65; − 9.82]	**< .001**	1.32

*WL* wait-list, *EMDR* eye movement and desensitization and reprocessing *PCL-5* PTSD Checklist for DSM-5, *BSI* Brief Symptom Inventory, *DT-P* Distress Thermometer for Parents, *OBVL* Parenting Stress Questionnaire. Significant *p* values < .05 are presented in bold

#### Secondary outcomes

Compared to wait-list, participants in the EMDR group reported significant reductions on total comorbid psychological symptoms (BSI), the distress thermometer and total distress score (DT-P), and parenting stress (OBVL) at T1/T.01 (Table 2). Effect sizes ranged from *d* = .63–1.83.

### Within-group comparisons (EMDR)

#### Primary outcome

Within-group comparisons (*N* = 14) showed significant reductions in total PTSD symptom severity and subscale scores (PCL-5) T1 vs. T0/T.01 (*d* = 1.04–2.16) and T2 vs. T0/T.01 (*d* = .96–2.30) (Table 3).

**Table 3:** Means and standard deviations pre-treatment (T0/T.01), post-treatment (T1), 3-months follow-up (T2), and within-group comparisons (EMDR)

	T0/T.01 (*N* = 14)	T1 (*N* = 13)	T2 (*N* = 12)	T0/T.01 vs. T1			T0/T.01 vs. T2		
	M (*SD*)	M (*SD*)	M (*SD*)	*B* [95% CI]	*p*	*d*	*B* [95% CI]	*p*	*d*
PCL-5									
Total score	40.21 (12.14)	16.54 (11.53)	14.33 (12.15)	− 24.44 [− 25.77; − 23.11]	**< .001**	1.95	− 24.94 [− 26.31; − 23.57]	**< .001**	1.98
Cluster B	9.71 (3.07)	3.08 (2.18)	2.42 (2.84)	− 6.84 [− 7.22; − 6.46]	**< .001**	2.16	− 7.25 [− 7.65; − 6.86]	**< .001**	2.30
Cluster C	4.43 (2.21)	1.31 (1.70)	1.33 (2.06)	− 3.30 [− 3.53; − 3.08]	**< .001**	1.04	− 3.30 [− 3.30; 2.83]	**< .001**	.96
Cluster D	13.42 (5.36)	6.00 (4.81)	5.67 (5.27)	− 7.76 [− 8.39; − 7.13]	**< .001**	1.39	− 7.30 [− 7.95; − 6.65]	**< .001**	1.31
Cluster E	12.64 (4.11)	6.15 (3.65)	4.92 (2.87)	− 6.54 [− 6.97; − 6.10]	**< .001**	1.61	− 7.32 [− 7.77; − 6.87]	**< .001**	1.80
BSI									
Total score	1.00 (.49)	.42 (.29)	.34 (.26)	− .57 [− .63; − .51]	**< .001**	1.12	− .62 [− .67; − .56]	**< .001**	1.21
DT-P									
Thermometer score	7.21 (1.58)	3.54 (2.96)	4.83 (2.41)	− 3.62 [− 3.99; − 3.24]	**< .001**	2.21	− 2.03 [− 2.42; − 1.64]	**.01**	1.24
Total problem score	20.93 (5.25)	11.00 (7.14)	11.33 (7.69)	− 10.09 [− 12.91; − 7.26]	**< .001**	1.87	− 9.07 [− 11.98; − 6.16]	**< .001**	1.69
OBVL									
Parenting stress total	87.00 (11.51)	70.92 (11.08)	74.58 (10.58)	− 16.60 [− 18.03; − 15.16]	**< .001**	1.43	− 11.32 [12.80; − 9.84]	**< .001**	.98

*WL* wait-list, *EMDR* eye movement and desensitization and reprocessing, *PCL-5* PTSD Checklist for DSM-5, *BSI* Brief Symptom Inventory, *DT-P* Distress Thermometer for Parents, *OBVL* Parenting Stress Questionnaire. Significant *p *values < .05 are presented in bold

#### Secondary outcomes

Within-group comparisons showed significant reductions in total comorbid psychological symptoms (BSI), the distress thermometer, total distress score (DT-P) and parenting stress (OBVL) T1 vs. T0/T.01 (*d* = 1.12–2.21) and T2 vs. T0/T.01 (*d* = .98–1.69) (Table 3).

#### Drop-out and adverse events

In total, all except one parent completed treatment (92%). One parent dropped out due to the COVID-19 lockdown after the first EMDR treatment day (7.14%) and missed two sessions EMDR in one half-day. Furthermore, one parent was lost to follow-up at T2. No adverse events were reported.

## Discussion

This is the first randomized clinical trial that investigates the feasibility and effectiveness of time-limited EMDR for traumatized parents of children with a progressive, life-limiting illness. We show that time-limited EMDR is feasible and effective in reducing the severity of PTSD symptoms, comorbid psychological symptoms, distress and parenting stress. These results are striking when taking into account the ongoing stressful events related to the progressive, life-limiting nature of MPS III.

Previous intervention studies concerning parental PTSD related to the child’s illness mainly focused on other treatment methods, including cognitive behavioral therapy, acceptance and commitment therapy and problem solving therapy [34–38]. The majority of these study concerned parents of children with cancer or cancer survivors and three out of five studies reported a decrease in PTSD symptoms after a range of 6–8 sessions. In comparison with these studies, time-limited EMDR in parents of children with MPS III shows a larger treatment effect in a shorter amount of time.

Effective treatment of parental PTSD is highly relevant for pediatric practice, as stress symptoms in parents impact on the psychosocial and physical wellbeing of the child. For example, PTSD may interfere with a responsive parenting coping style or the ability to provide the necessary medical care to the child [39, 40]. Although earlier studies showed elevated rates of PTSD symptoms in parents of children with e.g., pediatric cancer, epilepsy or burn injuries [6, 41, 42], literature on parents of children with neurodegenerative, life-limiting illnesses as well as on treatment of PTSD in this specific population is lacking. This might be due to the fact that screening and treatment of PTSD is less often considered in pediatric practice when parents are confronted with ongoing, long-term child-related traumatic experiences. However, parents who perceive a continuous risk of life threat related to their child’s illness have the highest risk to suffer from PTSD [43]. Based on the large effect sizes and persistence of the effects found for EMDR in our trial we feel that this treatment should be offered to parents, especially in the presence of ongoing traumatic events. Regular monitoring of these parents for PTSD symptoms seems warranted and it is plausible that trauma-focused treatment may be indicated more than once during the course of the disease.

The response rate to invitation in the current trial was relatively low. In a previous study including 45 parents of MPS III patients we showed that at least 80% of the parents experience at least one PTSD symptom and 22% met the criteria for a probable PTSD diagnosis [12]. The response rate in our study may reflect the high burden of parenting a child with a life-limiting illness leading to less time for prioritizing of own (psychosocial) health [2]. Moreover, parents possibly do not associate their distress with unprocessed stressful experiences and might be unfamiliar with EMDR therapy. Also, inclusion of parents started shortly before the COVID-19 pandemic reached the Netherlands. Many parents mentioned the extra burden on their family as result of e.g., closing of medical daycares, combining the care for their child with homeschooling of siblings, and financial distress. These additional stressors might have negatively impacted the response rate. Due to the COVID-19 pandemic, therapists has started to offer EMDR online, which could be a promising approach to lower the barrier for parents to receive trauma treatment in the future [44].

Some limitations of the current study should be discussed. First, this study was initially named as pilot study (https://trialsearch.who.int/Trial2.aspx?TrialID=NL8496) due to the small sample size, and statistical outcomes should be interpreted with caution. Since all families of living MPS III patients in the Netherlands were approached for this study and the effects of EMDR therapy are large, we consider our study of sufficient evidence to recommend EMDR for this specific population. Second, the generalizability of the findings may be limited. However, we assume that this treatment may also be beneficial for parents of children with other neurodegenerative life-limiting illnesses. Finally, the lack of a long-term follow up measurement limits conclusions about the maintenance of the treatment effects.

## Conclusions

In conclusion, this study shows that time-limited EMDR therapy is a feasible and effective treatment for traumatized parents of children with a rare progressive life-limiting illness. Recurrent monitoring for PTSD symptoms in parents, and, if needed, offering this treatment should be introduced in everyday pediatric practice.

## Supplementary Information

**Additional file 1:** Supplementary material A.

## Data Availability

Data that support the findings of this study are available from the corresponding author on reasonable request.
