# Supplementary material for: Effectiveness of time-limited eye movement desensitization reprocessing therapy for parents of children with a rare life-limiting illness: a randomized clinical trial
Source: Orphanet J Rare Dis. 2022 Sep 2;17:328. doi: 10.1186/s13023-022-02500-9 (PMC9437394; doi:10.1186/s13023-022-02500-9)
Supplement: Supplementary file 1 — Additional file 1: Supplementary material A. [file 13023_2022_2500_MOESM1_ESM.docx]

**Supplementary material A**

*Calculation of effect sizes*

Between group effect sizes were calculated by subtracting the estimated mean difference for pre-to-post wait-list (WL) from the pre-to-post EDMR and dividing the mean difference by the pooled baseline standard deviation. Within group effect sizes were calculated by dividing the estimated difference in means from T0/T.01 to T1 and T0/T.01 to T2 by the pooled baseline standard deviation.
